# Hansemann’s anaplastic theory of cancer after 135 years

**DOI:** 10.3389/freae.2025.1607433

**Published:** 2025-05-02

**Authors:** Steven Henikoff, Kami Ahmad

**Affiliations:** ^1^ Basic Sciences Division, Fred Hutchinson Cancer Center, Seattle, WA, United States; ^2^ Howard Hughes Medical Institute, Chevy Chase, MD, United States

**Keywords:** chromosome, centromere, anaplasia, aneuploidy, histones, CENP-A

## Abstract

Summary: The term “anaplasia” was coined in 1890 to describe chromosomal changes common to primordial cancer cells, but ever since the mechanisms whereby a cell becomes anaplastic has been the subject of much speculation. Recent results based on genomic and epigenomic profiles of cancer patient samples provide a glimpse into early events that lead to aneuploidy, the original defining feature of an anaplastic cell. We propose that the anaplastic cell is one in which RNA Polymerase II hypertranscribes S-phase-dependent histone genes, and the resulting histone excess facilitates DNA replication while competing for CENP-A, causing centromere breaks that initiate whole-arm aneuploidy.

During the last decade of the 19th century, David Hansemann described striking chromosomal abnormalities in many different types of epithelial cancers including asymmetric mitoses (Hansemann, 1890) and trivalent spindles (Hansemann, 1891). In his many influential publications on what was later called aneuploidy (Bignold et al., 2007), Hansemann laid the groundwork for the modern view of cancer, in which a tumor develops from a single “anaplastic” de-differentiated cell that acquires independent existence within a tissue composed of “altruistic” cells that differentiate normally. Although Hansemann’s ideas were initially met with skepticism, they were later modified and popularized by the embryologist Theodor Boveri after the rediscovery of Mendel’s laws (Boveri, 1914). However, what constitutes “anaplasia”, and whether it has a genetic, an epigenetic or an environmental trigger, remains speculative. Boveri imagined an abnormal environmental disturbance of a primordial cancer cell that results in spindle abnormalities and chromosome mis-segregations, and the idea that mitotic errors underlie aneuploidy has become entrenched in the field. For example, reviews of aneuploidy invariably describe merotelic attachments, extra centrosomes, unattached kinetochores or cohesion defects as driving aneuploidy, without addressing the question of what triggers these defects in an altruistic cell to become anaplastic (Ben-David and Amon, 2020; Gordon et al., 2012). The idea that whole-chromosome or whole-arm abnormalities drive cancer (Duesberg, 2007) has been clarified by recent work describing how changes in copy numbers of cancer driver and tumor suppressor genes resulting from aneuploidy alter the fitness landscape (Shih et al., 2023). However, it is unclear how therapeutic strategies to prevent aneuploidy might be designed given the plethora of mechanistically unrelated mitotic defects. But if we can understand how anaplasia leads to aneuploidy, then we might envision practical anti-aneuploidy therapies.

Among the forms that Hansemann illustrated were examples of chromatids away from the metaphase plate that were either attached or unattached to the mitotic spindle, in addition to many examples of multipolar spindles (Figure 1). Cytological analysis methods, such as fluorescence *in situ* hybridization (FISH), revealed that some aneuploidies were specific to particular cancer types, and could be used to predict outcome. For example, loss of chromosome arm 5q in acute myeloid leukemia predicts a more positive outcome after bone marrow transplantation, while loss of chromosome 7 or arm 7q predicts a more negative outcome (Deeg et al., 2012). More recently, genomics-based methods have been used to score aneuploidy. For example, the Cancer Genome Atlas (TCGA) project developed an aneuploidy score based on analysis of whole-genome sequencing data across 33 cancer types (Taylor et al., 2018). This analysis revealed that overall ∼80% of aneuploid chromosomes were whole-arm gains or losses, whereas the other ∼20% were losses or gains of both p and q arms typical of whole-chromosome losses (Zheng et al., 2025), with frequent changes in genome ploidy (Prasad et al., 2022). Expansions of aneuploid epithelial cells, including autosomal whole-arm gains and losses, have recently been described in normal breast tissues from all 49 healthy women tested (Lin et al., 2024), compelling evidence that anaplastic cells arise frequently from among altruistic cells as Hansemann originally envisioned 135 years ago. These aneuploid expansions in normal breast included gain of 1q and losses of 10q, 16q and 22q, which are frequent in invasive breast tumors, presumably under selection for oncogene gain or tumor suppressor loss (Shih et al., 2023). For example, deficiency of the NF2 tumor suppressor gene on 22q results in activation of the YAP1 protein, which can lead to cancer cell proliferation via the Hippo signaling pathway (Szulzewsky et al., 2022). Such observations of anaplastic cells with a selective advantage over surrounding normal cells raise the question of what initiates whole-arm aneuploidy?

**FIGURE 1:**
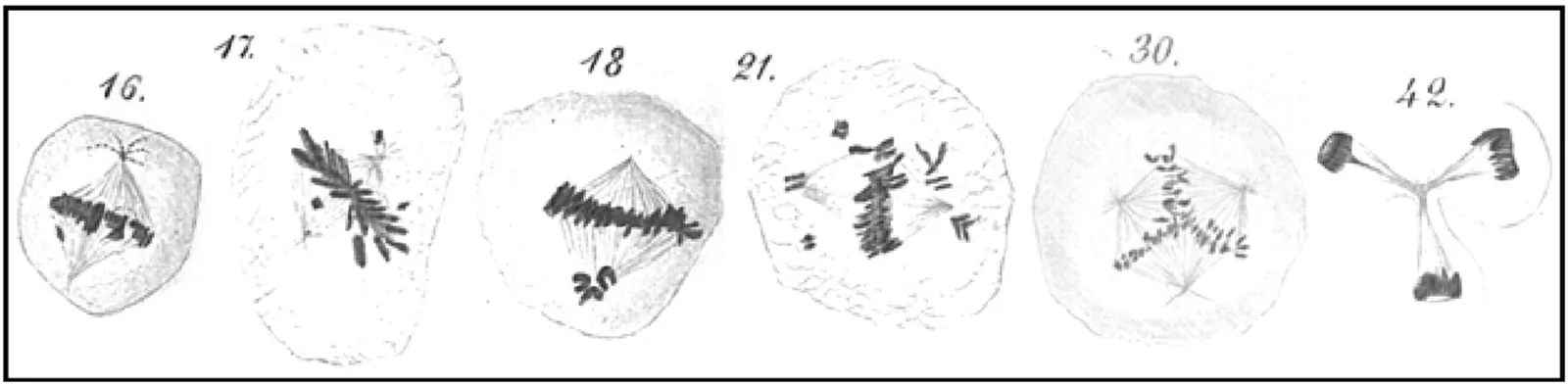
Aneuploidy in cancer. Drawings of aneuploid mitotic figures by David Hansemann illustrating examples of whole-chromosome mis-segregations and multipolar spindles. Reproduced from Hansemann (1891).

The generation of a chromosome arm from a metacentric chromosome requires a break at the centromere, so the preponderance of whole-arm gains and losses means that the large majority of aneuploid chromosomes in a cancer cell must have been generated by centromere breaks. Centromere breaks can occur upon depletion of the histone H3 variant CENP-A, the essential epigenetic foundation of the centromere (Giunta et al., 2021; Figure 2). These breaks evidently result from conflicts between the DNA replication apparatus and RNA Polymerase II (RNAPII) while it is transcribing centromeric alpha-satellite repeats during S-phase. Centromere breaks during S-phase resulting in gains or losses of whole chromosome arms provide an alternative to the traditional mitotic error explanation for aneuploidy in cancer. Indeed, the possibility of centromere breaks caused by mitotic events such as merotelic attachments is highly unlikely to account for the high frequency of aneuploidy in cancer given the amount of force that would be required (Bloom, 2008) and the mechanisms that have evolved to prevent centromere breaks at anaphase (Cimini, 2023).

**FIGURE 2:**
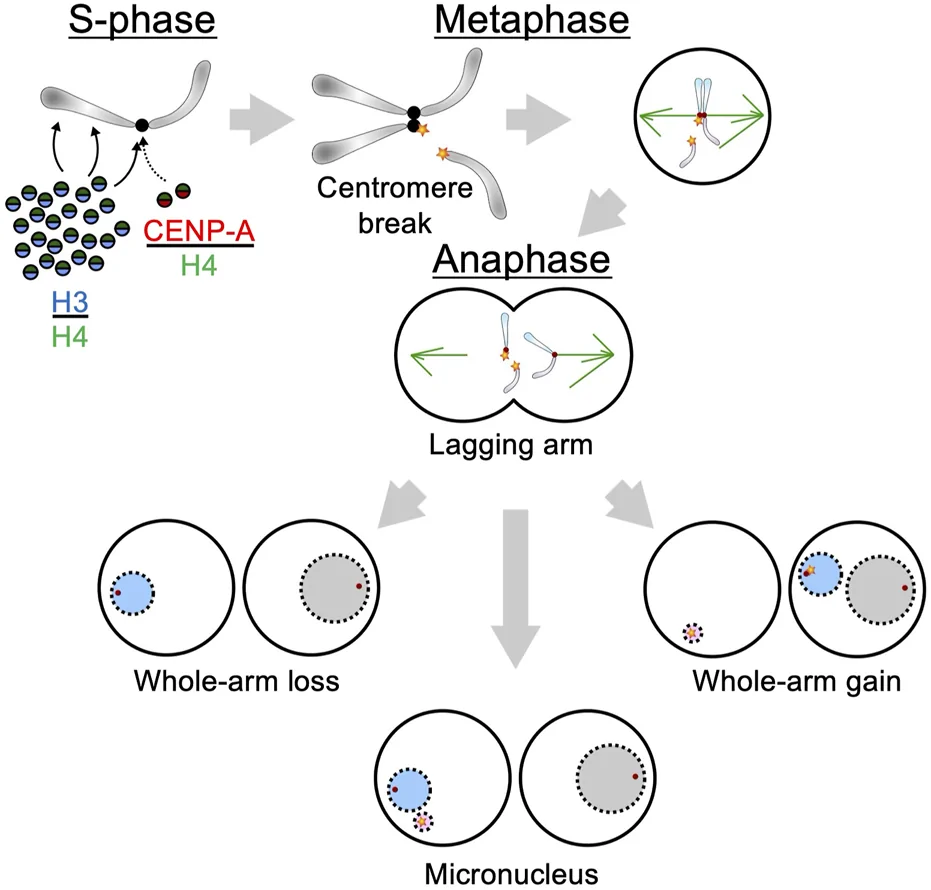
Model for generation of aneuploidy resulting from centromere breaks. Overproduction of S-phase-dependent histones promotes both anaplasia and chromosome instability. Excess histone H3/H4 dimers compete with centromeric histone dimers (CENP-A/H4) during S-phase, and displacement causes breaks, leading to widespread aneuploidy (blue) through whole chromosome arm loss, arm gain, and through whole chromosome loss. The occurrence of micronuclei by encapsulation of fragmented chromosome arms further stimulates chromosomal instability.

As histone H3 competes with CENP-A for incorporation at centromeric alpha-satellites (Blower et al., 2002), one potential mechanism for generating centromere breaks is excess histone production. Hypertranscription, defined as the global upregulation of gene expression, is observed in most cancers (Zatzman et al., 2022), and we have very recently shown that RNAPII hypertranscription of histone genes accurately predicts recurrence in non-invasive, mostly benign, meningiomas (Henikoff et al., 2025). We also observed that RNAPII abundance at histone genes correlated with aneuploid whole-arm losses over gains for 38 of the 39 autosomal whole arms, which was unexpected given the general perception that *specific* whole-arm gains and losses should drive specific cancers. When we applied the same analysis to RNAPII data from invasive breast cancers, we again found that abundance over histone genes correlated with aneuploid whole-arm losses over gains for 38 of 39 autosomal whole arms. Total whole-arm losses in excess of gains also predicted recurrence based on RNA-sequencing data from 1298 patient meningiomas and combined whole-genome sequencing data from 10,522 patient tumors from 33 cancer types catalogued in TCGA (Zheng et al., 2025). Taken together, our results suggested that excess histone H3 competes with CENP-A for incorporation into centromeric alpha-satellites, causing centromere breaks and whole-arm losses, driving aneuploidy without requiring additional errors during mitosis. Less frequent whole-arm gains might result from encapsulation of lagging whole arms within micronuclei, and replication followed by attachment to the broken centromere at the next metaphase for three total copies (Figure 2). This new paradigm for aneuploidy in cancer opens up the possibility of therapeutic regimens to prevent aneuploidy by targeting the unique machinery that produces S-phase-dependent histone mRNAs (Ahmad et al., 2024).

In conclusion, our evidence for histone overproduction leading to centromere breaks provides a mechanistic model for the generation of aneuploidy in cancer, although it still leaves open the question of what triggers histone overexpression and whether it is genetic, epigenetic or environmental. During S-phase, histone genes are activated by Cyclin E/CDK2 phosphorylation (Geisler et al., 2023), and at least in *Drosophila* are bound by the Myc transcription factor (Daneshvar et al., 2011), and perhaps rare, transient bursts of these known cancer drivers initiate the process. Subsequent generalized aneuploidy–which is known to inhibit cellular differentiation and stimulate proliferation–will predispose cells to anaplasia and cancer.

## Data Availability

The original contributions presented in the study are included in the article/supplementary material, further inquiries can be directed to the corresponding author.
